# Multiple metallic-shell nanocylinders for surface-enhanced spectroscopes

**DOI:** 10.1186/1556-276X-6-173

**Published:** 2011-02-24

**Authors:** Jin-You Lu, Kuo-Pin Chiu, Husan-Yi Chao, Yuan-Huei Chang

**Affiliations:** 1 Department of Physics, National Taiwan University, Taiwan; 2 Department of Physics, Chung Yuan Christian University, Taiwan

## Abstract

The optical properties of multiple dielectric-core-gold-shell nanocylinder pairs are investigated by two-dimensional finite difference time domain method. The core-shell cylinders are assumed to be of the same dimension and composition. For normal incidence, the diffraction spectra of multiple cylinder pairs contain the lightning-rod plasmon mode, and the electric field intensity is concentrated in the gap between the nanocylinder pairs in the infrared region. The resonance wavelength and local field enhancement of this plasmon mode can be tuned by varying the pair-distance between the pairs, the gap-distance between the pairs, and the optical constants of the dielectric-core and the surrounding medium. The results show that the multiple core-shell nanocylinder pair contains the plasmon mode same as that of the solid metallic cylinder pairs at the long wavelength part of the spectrum. The large electric field intensity in the infrared region at long wavelength makes multiple core-shell cylinders as ideal candidates for surface-enhanced spectroscopes.

## Introduction

In the emerging field of plasmonics, dielectric-core-metallic-shell nanostructures have attracted much attention. Compared to solid metallic particles, these core-shell nanostructures exhibit highly tunable plasmon modes that can be tuned over an extended wavelength range between visible and near-infrared regions. The variation of the plasmon resonance wavelength is interpreted as originating from the coupling of localized surface plasmon modes at the inner and outer surfaces of the core-shell structure [1,2]. The dependence of their optical properties on the size, shape, and surrounding medium is an active subject of research, and recent advances in nanofabrication have enabled us to design nanostructures with different shapes and functionalities, such as nanorices [3], nanorings [1], and nanoshells [4].

The most widely used surface-enhanced spectroscopy is surface-enhanced Raman scattering (SERS) [5,6], where the electric field intensity on the molecule is required to be maximized to enhance the detected signals. Such an enhancement can be very large in the gap region between two closely spaced nanoparticles [7-9]. The Raman enhancement is approximately given by ${\left|{\vec{E}}_{\text{loc}}/{\vec{E}}_{\text{inc}}\right|}^{4}$, where ${\vec{E}}_{\text{loc}}$ is the local electric field near the detected molecule, and ${\vec{E}}_{\text{inc}}$ is the incident electric field. For core-shell dimmers [10-12], the dramatically enhanced electric field and tunability in the resonant frequency can be obtained by varying the geometrical parameters. The same effect can also be used to enhance infrared signals and is called the surface-enhanced infrared absorption (SEIRA) spectroscopy [13]. This enhancement is not as strong as for Raman spectroscopy in the visible range, but the enhancement factors are still proportional to the square of the electromagnetic field. Originally, the local field enhancement of surface plasmon excitations in plasmonic nanostructures can only be found in the visible and UV regions, which is suitable for SERS. Recently, nanoshell arrays have been found to possess ideal properties as a common substrate for both SERS and SEIRA spectroscopies. The reason for this is that nanoshell arrays can provide large electric field enhancement at the same spatial location in both the visible and infrared regions of the spectrum [14].

In this article, the plasmon modes of multiple dielectric-core gold-shell cylinder pairs for normal incident light are investigated. The simulation results show that the multiple nanocylinder pairs provide large electromagnetic enhancement in the wide spectral range between visible and infrared regions. The localized surface plasmon excitation occurring in the infrared region in the spectrum is due to the efficient metallic screening effect at low frequency. The infrared plasmon resonance is identified as due to the interactions between the electrons at the outer metallic surfaces of the nanocylinder pairs, which is independent of the electrons at the inner surfaces. The other plasmon modes of multiple nanocylinder pairs are only weakly red-shifted from the individual nanocylinder pair plasmon modes. This analysis shows how to tune the plasmon modes of multiple nanocylinder pairs by varying the pair-distance between the pairs, the gap-distance between the pairs, and the optical constants of the dielectric-core and surrounding medium.

When the number of the cylinder pairs is three, it is found that the lightning-rod effect results in two plasmon modes in the infrared region. One of the two infrared plasmon modes is similar to the open cavity mode of three solid metallic nanocylinder pairs studied by Wu [15,16]. The strongest electric field enhancement of such a cavity mode exhibits in the gap of the second pair and the open cavity has a linear relation between the resonant wavelength and the radius of nanocylinders. Therefore, the multiple core-shell nanocylinder pair contains the plasmon mode same as that of the solid metallic cylinder pairs at the long wavelength part of the spectrum.

## Calculation methods

Our finite difference time domain (FDTD) simulation domain is divided into three regions and they are, from outside to inside, absorbing boundary, scattered field region, and the total field region. The convolution perfectly matched layers, which absorb electromagnetic field efficiently, are used as absorption boundary to prevent reflections of scattered waves back into the simulation domain. The FDTD calculations were performed accurately by using a mesh size of 0.5 nm and a Courant number of 0.5. The program codes have been checked with the analytic theory [17]. The optical response of gold is modeled using the three critical point pole pairs (CP3) model [18], which provides a good fit to the tabulated experimental data [19]. The far-field responses for extinction, absorption, and scattering can be calculated from the Poynting vector [20]. The absorption cross section can be obtained by integrating the inward-going component of the Poynting vector of the total field over the surface *S*,

Cabs=−12∮SRe(E→×H→*)⋅n∧dsP0,

where *P*_0 _is the incident power. The scattering cross section is calculated by integrating the Poynting vector of the scattering field over the surface *S*:

Csca=12∮SRe(E→s×H→s*)⋅n∧dsP0.

With some substitutions and rearrangements, the extinction cross section, which is the sum of the scattering and absorption cross sections, can be written:

Cext=−12∮SRe(E→×H→inc*+E→inc*×H→)⋅n→dsP0,

where inc in the subscript means incident fields. In addition, the near-field electric field intensity ${\left|\vec{E}\right|}^{2}/{\left|{\vec{E}}_{\text{inc}}\right|}^{2}$ is the electric field distribution normalized by the incident continuous electric wave. In this simulation, the cylinder is of the same geometry and composition. The inner and outer radii of the dielectric-core-gold-shell nanocylinder are 60 and 80 nm, respectively; the propagating direction of the incident wave is along the axis that connects the nanocylinder pairs, and the polarization of the incident wave is parallel to the axis that connects the two nanocylinders of the pair.

## Results and discussion

**Figure 1:**
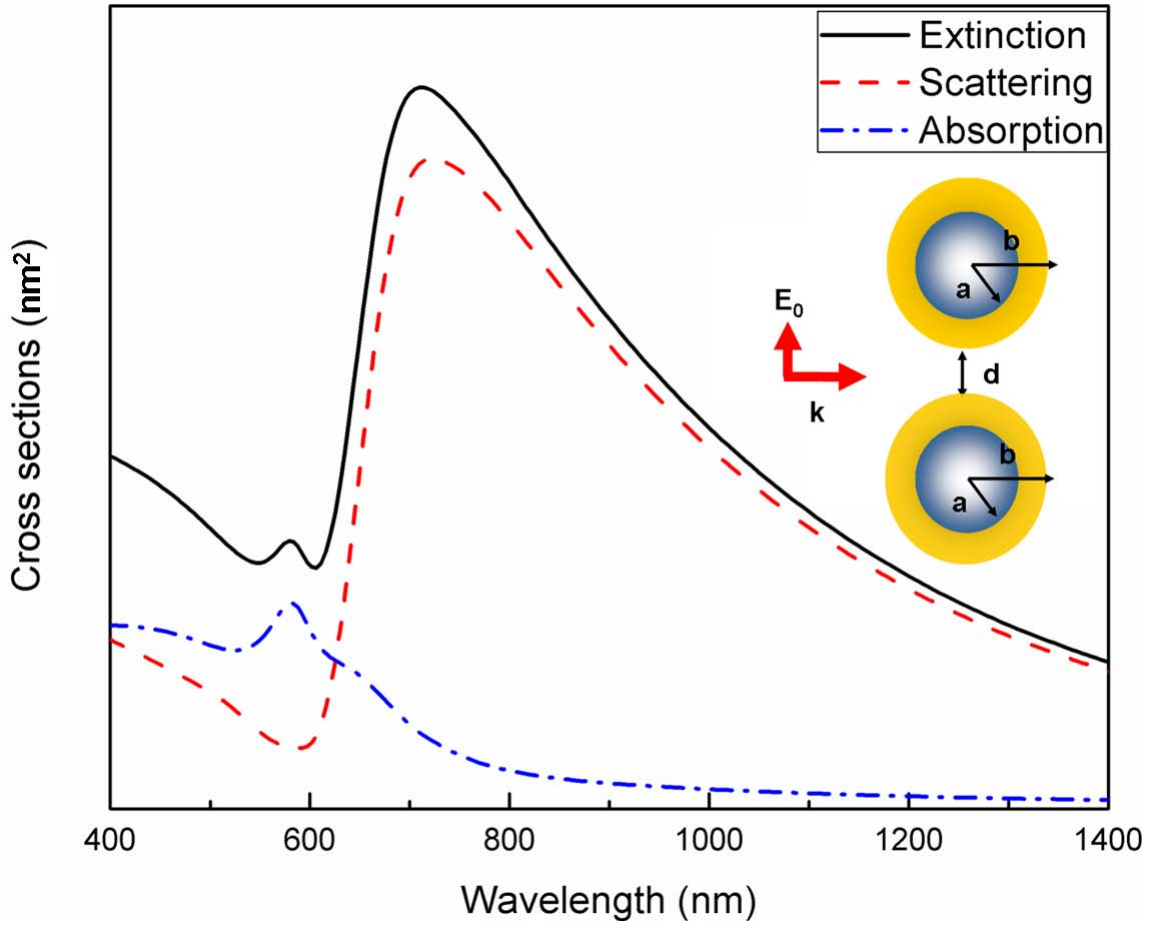
**The FDTD-calculated extinction, scattering, and absorption spectra of a dielectric-core-gold-shell nanocylinder pair with a separation distance of 20 nm**. The permittivity of the dielectric core is 2.1. The light incident geometry is shown in the inset.

The spectral characteristics of a single dielectric-core-gold-shell nanocylinder pair are first investigated. The geometry of the nanocylinder is shown in the inset of Figure 1, and the distance between the nanocylinders is 20 nm. The dielectric constant of the dielectric core inside the nanocylinder is 2.1, which is the dielectric constant of the silica. From Figure 1, it can be seen that the extinction spectrum is characterized by two plasmon modes. The plasmon mode, of approximately 700 nm, corresponds to in-phase symmetric dipole-dipole interaction mode, and the symmetric dipole mode is a result of the electrons at the inner surface of the core-shell nanocylinder aligned symmetrically with the electrons at the outer surface [21].

The other plasmon mode, of approximately 575 nm, corresponds to in-phase symmetric quadrupole-quadrupole plasmon mode. The out-of-phase plasmon mode cannot not be observed in the spectrum. This is because out-of-phase plasmon modes do not contain any the dipole moment, so that external light cannot excite these modes.

**Figure 2:**
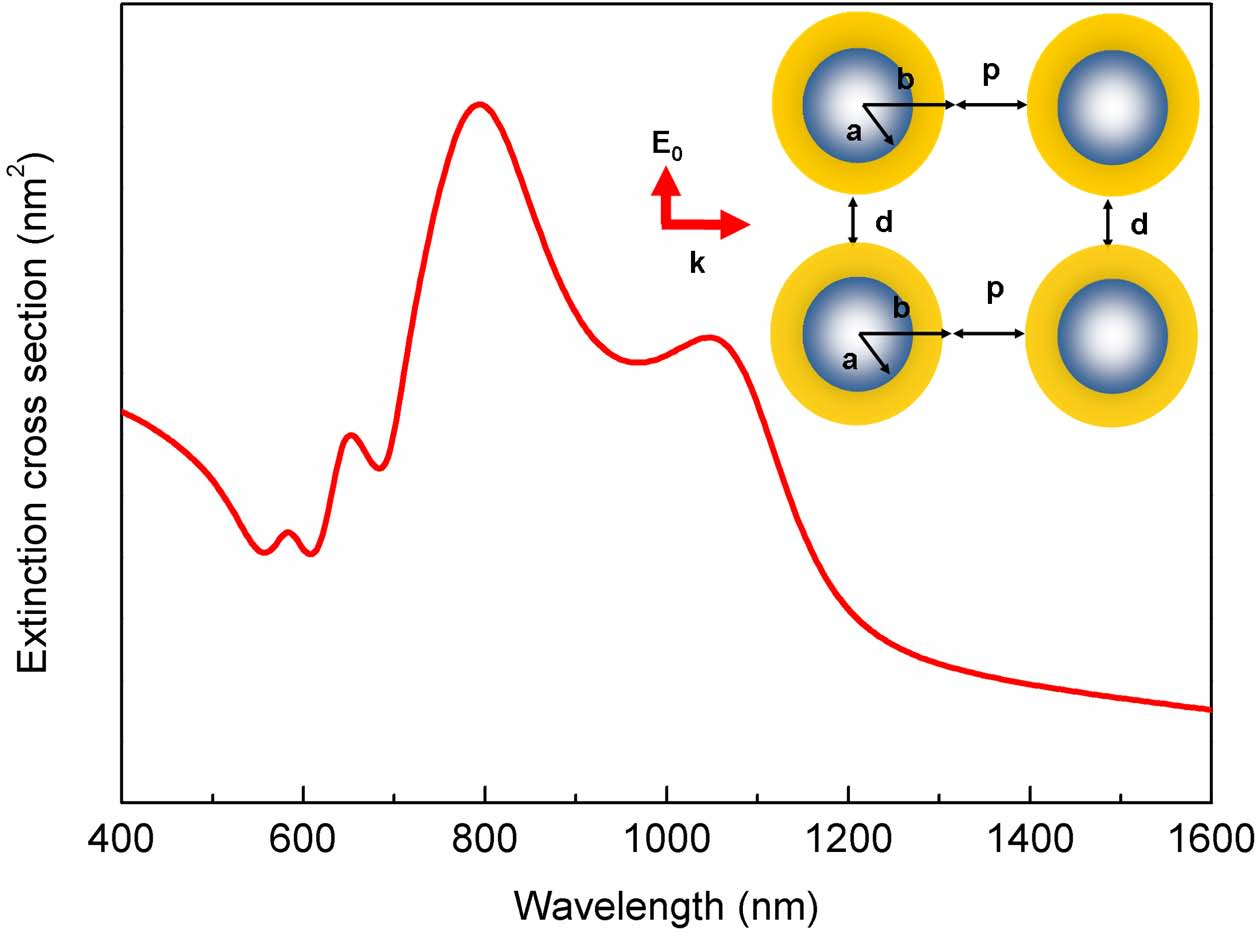
**The extinction spectra of two silica-core gold-shell nanocylinder pairs with the gap width of 20 nm and pair-distance of 20 nm**.

When two core-shell nanocylinder pairs are brought together with the pair distance *p *of 20 nm, the interaction between the two nanocylinder pairs is strong and results in the energy splitting in the spectrum. The other parameters are the same as those shown in Figure 1. The plasmon modes of two nanocylinder pairs are unveiled in Figure 2, which shows four major plasmon modes in the spectra between the visible and infrared regions. The plasmon mode, of approximately 800 nm, corresponds to the two in-phase symmetric dipole-dipole modes oscillating in the out-of-phase way. In other words, when two nanocylinder pairs couple with each other, the in-phase symmetric dipole mode of a single dielectric-core-gold-shell interacts with that of another nanocylinder pair. Phase retardation effect results in the out-of-phase oscillation for the two in-phase symmetrical dipole-dipole modes [22].

The plasmon mode of approximately 690 nm is produced from the interaction between in-phase dipole-dipole mode of the first nanocylinder pair and the in-phase quadrupole-quadrupole mode of the second nanocylinder pair. The other Plasmon mode, of approximately 620 nm, corresponds to two in-phase quadrupole-quadrupole modes oscillating in the out-of-phase way. Therefore, for normal incidence, the plasmon modes of two nanocylinder pairs are composed of the individual nanocylinder pair plasmon mode.

**Figure 3:**
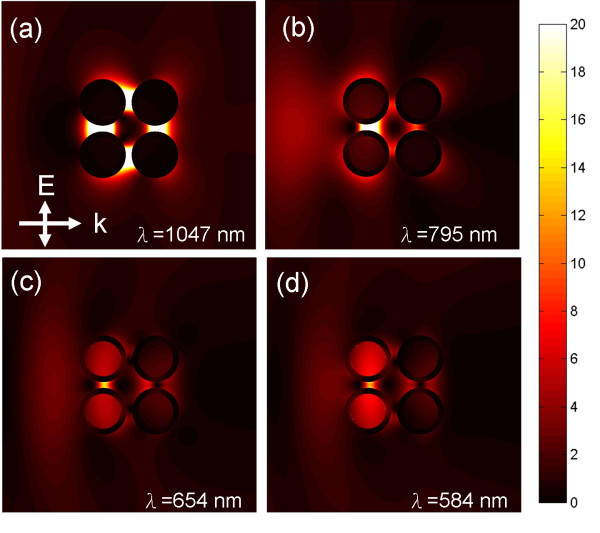
**The normalized electric field intensity at the respective coupling plasmon modes wavelength of two silica-core gold-shell nanocylinder with the same geometry shown in Figure 2**.

In addition, it is found that one of the plasmon modes is excited at much longer wavelengths. The electric field distribution shows that this mode is the result of the interaction between the electrons at the outer surface of the four nanocylinders. The normalized electric field intensity is investigated by propagating a plane wave at the respective plasmon modes of two dielectric-core-gold-shell nanocylinder pairs with the same geometry as shown in Figure 3. It is found that the electric field intensity decreases inside the dielectric core of the nanocylinder when the incident wavelength increases. This is due to the metal screening effect because the skin depth of gold increases when the wavelength of incident wave decreases.

When the incident wavelength increases to 1050 nm, the incident wave cannot penetrate into the nanocylinder. The maximum electric field intensity concentrates in the gap between the nanocylinders, and such a phenomenon is called lightning-rod plasmon mode [23]. When the electric field is completely screened by the metal, no electric field and no induced charges could be found in the inner side of the metallic shell. This plasmon mode is the effect of the coupling of the in-phase dipolar modes formed by the electrons at the outer surfaces of the core-shell nanocylinders. From Figure 3, it can be known that except for this plasmon mode of approximately 1050 nm, other plasmon modes consist of the essential plasmon modes of the core-shell nanocylinder pair. The lightning-rod plasmon mode for a single dielectric-core-gold-shell nanocylinder pairs cannot be observed. This is because for just a single core-shell nanocylinder pair with the outer radius of 80 nm, the resonance wavelength of the in-phase dipolar mode resulted from the outer surfaces does not exist in the infrared region [24]. When two core-shell nanocylinder pairs interact with each other, such a plasmon mode can be observed. The lightning-rod plasmon mode is red-shifted to longer wavelength than those of plasmon modes that are a result from coupling of the individual nanocylinder pair plasmon modes due to the pair-pair interaction.

**Figure 4:**
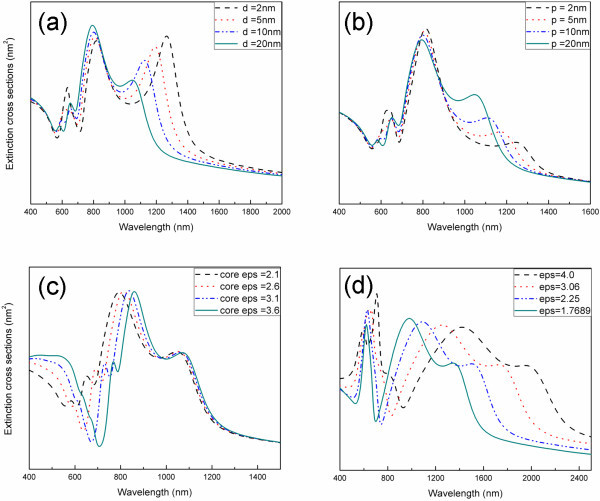
**The dependence of the extinction spectra of two dielectric-core-gold-shell nanocylinder pairs on dimensions**. **(a) **For different gap widths between the nanocylinders with the pair distance *p *of 20 nm, **(b) **for different pair-distances between the nanocylinder pairs with the gap width of 20 nm, **(c) **for different dielectric constant of the dielectric cores, **(d) **for different surrounding medium.

In the following sections, it is shown that the plasmon modes of the two core-shell nanocylinder pairs can be tuned systematically by varying the gap-width d between the nanocylinder pair, pair-distance *p *between the pairs, the dielectric constant of the dielectric core inside the nanocylinder, and the surrounding medium. First, the two pairs are simulated by varying the gap-distance d between the nanocylinder pair with the fixed pair-distance of 20 nm as shown in Figure 4a. As the inter-cylinder distance decreases, the lightning-rod plasmon mode is red-shifted and enhanced due to the much stronger interaction of dipolar modes as a result of the electrons at the outer surface of the nanocylinders. As the intensity of this plasmon mode increases, a dramatically larger electric field concentrates in the gap of the nanocylinder pair. As for other plasmon modes that are a result of the interactions of the individual core-shell nanocylinder par plasmon modes, the resonance wavelength and intensity almost remain the same. This is because the cavity mode inside the dielectric core was formed due to the sufficient effective length, which is similar to the one-dimensional Fabre-Perot resonance mode. (Therefore, these plasmon modes associated with the cavity mode do not change dramatically by outside of the nanocylinders.)

Figure 4b shows that as pair-distance decreases between the core-shell cylinder pairs, the intensity of the plasmon mode, of approximately 1100 nm, decreases and is red-shifted. The resonance energy shifts to lower energy because the two cylinder pairs oscillate in the out-of-phase way. As the pair-distance decreases, the static coulomb energy also decreases [22]. The interaction between the cylinder pairs gathers the energy flow inside the region between the pairs and this interaction also enhances the backscattering effect [25]. However, the enhancement of the backscattering results in the reduction of the lightning-rod effect because the amount of the incident energy flow concentrating in the gap of the nanocylinder pair decreases.

The two nanocylinder pairs with different dielectric constants of dielectric cores are also simulated as shown in Figure 4c. The figure shows that as the refractive index of the dielectric core increases, the resonance energy of the plasmon modes reduces, except for the lightning-rod plasmon mode. This is because for the core-shell nanocylinder pairs, lightning-rod plasmon mode is only the result of the electrons at the outer surface of the nanocylinders. A large real permittivity of the dielectric core results in an efficient screening and thus results in a redshift of the plasmon energies [26,27]. The resonance energy of other plasmon modes associated with the electrons of the inner surface of the nanocylinders can be decreased by increasing the refractive index of the dielectric core. Similarly, as the refractive index of the surrounding medium is increased as shown in Figure 4d, the resonance energy of all the plasmon modes will decrease. This is because all the plasmon modes are dependent on the electrons at the outer surface of the nanocylinders.

**Figure 5:**
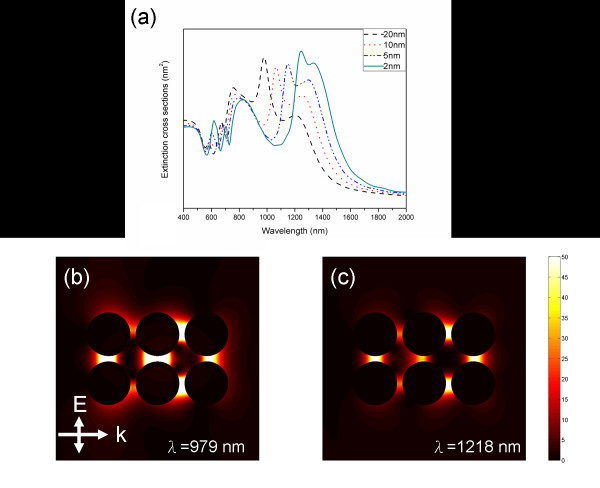
**The dependence of the extinction spectra of three dielectric-core-gold-shell nanocylinder pairs on dimensions**. **(a) **The dependence of the extinction spectra on the pair distance between the three nanocylinder pairs. **(b) **For the pair distance of 20 nm, the electric field intensity at the wavelength of 979 nm. **(c) **For the pair distance of 20 nm, the electric field intensity at the wavelength of 1218 nm.

The simulation results of the three nanocylinder pairs with different pair-distances *p *from 20 to 2 nm are shown in Figure 5a, and the spectra become more complex. Two lightning-rod plasmon modes can be found in the infrared range in the extinction spectra and the resonance wavelengths are 979 and 1218 nm, respectively. From the electric intensity of these two plasmon modes shown in Figure 5b, c, one can be convinced that the two plasmon modes are due to the dipolar coupling of three solid metallic pairs but with different charge distributions. A very interesting situation occurs when the wavelength of the incident light is 979 nm, in which case the maximum electric field intensity occurs in the gap of the second pair. Ng and Liu [15,16] pointed out that a cavity mode confined by three nanocylinder pairs can be found in the plasmonic spectra of the three-pair metallic nanocylinders. In this experiment, it is found that such a cavity mode of three solid nanocylinder pairs is essentially the same as the lightning-rod mode of approximately 979 nm. Therefore, the multiple core-shell nanocylinder pairs show higher tenability than those of the solid nanocylinder pairs and the properties of the lighting-rod plasmon mode of three core-shell nanocylinder pairs are known

From the simulation results discussed, one can understand how to control the lightening mode and other plasmon modes which are resulted from the interaction between the individual core-shell nanocylinders in the multiple nanocylinder pairs. Multiple nanocylinder pairs can provide the enhancement of the electric field intensity on the gap between the nanocylinder pair in the spectra from the visible to the infrared region. Therefore, they efficiently combine two SERS and SEIRA substrates on a single substrate.

## Conclusion

In conclusion, the plasmonic properties of multiple nanocylinder pairs are investigated by the FDTD method. The interaction between the dipolar mode of the nanocylinder results in the lightning-rod plasmon mode in the infrared region. The interaction between the essential plasmon modes was due to a single core-shell nanocylinder pair that resulted in other plasmon modes of multiple nanocylinder pairs. One can systematically control the strength and resonance energy of these plasmon modes by varying the pair-distance between the pairs, the gap-distance between the pair, the optical constant of the dielectric-core, and the surrounding medium. The multiple core-shell nanocylinder pair contains the plasmon mode same as that of the solid metallic cylinder pairs at the long wavelength part of the spectrum due to metallic screening effect. In particular, one of the lightning plasmon modes in three core-shell cylinder pairs is similar to the cavity mode confined by three solid cylinder pairs. Therefore, the core-shell nanocylinder pairs possess an ideal property that can enhance the electric field intensity at the same spatial positions in the wide wavelength range between the visible and the infrared regions.

## Abbreviations

FDTD: finite difference time domain; SEIRA: surface-enhanced infrared absorption; SERS: surface-enhanced Raman scattering.

## Competing interests

The authors declare that they have no competing interests.

## Authors' contributions

JY carried out the simulation work, performed the analysis, and drafted the manuscript. KP participated in the design of the study. HY participated in the design of the study. YH performed the analysis and made the corrections to the manuscript.
